# Irish secondary school science teachers’ perspectives on addressing the COVID-19 crisis as socioscientific issues

**DOI:** 10.1186/s43031-022-00056-z

**Published:** 2022-04-25

**Authors:** Ruth Chadwick, Eilish McLoughlin

**Affiliations:** CASTeL & School of Physical Sciences, Dublin City University, Dublin, Ireland

**Keywords:** Secondary education, Science, Socioscientific issues

## Abstract

Development of scientific literacy is a crucial aim of science education across the globe and research suggests that this can be realized through student exploration of socioscientific issues. While the COVID-19 crisis, emergency school closures and restrictions to in-class teaching, had negative impacts on teaching and on student learning and wellbeing, it also presents an opportunity to explore authentic socioscientific issues. This research explores teachers’ perspectives on addressing the COVID-19 crisis as socioscientific issues in secondary science education. This qualitative study surveyed 266 Irish secondary school science teachers about their experiences during the COVID-19 crisis. Thematic analysis was used to identify the reasons why teachers did and did not address the COVID-19 crisis as SSI. These findings were triangulated with findings from follow-up interviews. The majority of teachers in this study addressed the COVID-19 crisis as SSI. The COVID-19 crisis was explored within the curriculum, through project work and research, and through classroom discussion. Teachers described four barriers to exploring the COVID-19 crisis with their students: The COVID-19 crisis was not part of the curriculum; The lack of F2F contact made judging students’ reactions challenging; There was already too much focus on the COVID-19 crisis in everyday life and concerns over student wellbeing while discussing the sensitive topic of the COVID-19 crisis. Teachers noted that addressing the COVID-19 crisis led to benefits to student learning, health, wellbeing and hygiene.

## Introduction

The development of scientific literacy is a crucial aim of science education across the globe, and research suggests its realization can be through student exploration of socioscientific issues (SSI) (Zeidler et al., 2019). SSI constitute scientific topics that stem from personal, local, national, and global contexts and combine scientific understanding with moral or ethical concerns (OECD, 2019; Zeidler & Nichols, 2009). SSI are controversial, meaning that they call for the exploration of a range of viewpoints stemming from differing interpretations of evidence (Oulton et al., 2004).

Learning experiences involving SSI use a wide range of teaching approaches that enable students to engage in research, surveys, and experimentation (Levinson, 2018). Students may explore SSI through classroom discussion, reasoning, and decision-making (Zeidler & Nichols, 2009). Classroom discussion of SSI allows students to build their understanding of the range of ideas and viewpoints held by others, such as their peers, and compare these views to their own. Secondary research into SSI exposes students to ideas from a broader range of sources that may vary widely from their own perceptions and experiences of the SSI (Zeidler et al., 2019).

Exploration of SSI develops knowledge and understanding, competencies and skills, and attitudes and values that contribute to scientific literacy (OECD, 2019; Zeidler et al., 2019). These authors state that engaging in these aspects promotes student understanding of the nature of science and supports students learning science content. Furthermore, these researchers note exploring SSI develops skills relating to reasoning and argumentation, perspective-taking, recognition of the contributions and limitations of science, and skepticism (Zeidler et al., 2019). Moreover, students can engage in socially responsible actions on the SSI within the classroom and their communities (Levinson, 2018). However, teachers addressing SSI encounter challenges when facilitating this exploration with their students. Studies indicate that taking part in limited and one-off, short-term investigation of SSI may not significantly impact students’ skills development. Zeidler et al. (2019) pinpoint that skills development is possible through longer-term, sustained engagement with SSI learning experiences. This requires planning on the part of the teacher (Bayram-Jacobs et al., 2019). These researchers also know that the most successful SSI should align with the curriculum, students’ interests, and teachers’ interests (Zeidler et al., 2019). OECD (2019) adds the consideration of personal, local, national, and global contexts to highlight the relevance of SSI to the students.

Practical constraints for teachers include lack of time within the school curricula and the lack of ready-made teaching materials as barriers to addressing SSI (Bayram-Jacobs et al., 2019; Chen & Xiao, 2020). Lack of pedagogical knowledge around SSI teaching also presents an obstacle for teachers (Bayram-Jacobs et al., 2019). SSI contexts are controversial topics, so there is an inherent risk of conflict or upset during classroom discussions. Teachers must use their pedagogical skills and knowledge to pre-empt these and deal with any that arise. These planning, teaching, and workload considerations require the teachers to be highly committed and willing to take risks (McCully et al., 1999). Regardless, Chen and Xiao (2020) reveal that teachers are keen to support SSI teaching within the curricula, but they are for prioritizing content knowledge acquisition over the investigation of SSI. The priority given to learning subject matter highlights the need to embed SSI-based inquiry within the curricular content thus providing time to implement (Bayram-Jacobs et al., 2019). These authors suggest a need for readily available or adaptable learning materials that relate the curricular aims to various SSI contexts.

The existing research shows the significance of engaging students in SSI-based inquiry in school science, the barriers to such a focus, and solutions to embed a societal dimension to learning science. Although there is awareness of the character of integrating SSI into science education, the research at hand is a large-scale study that will explore Irish secondary science teachers’ perspectives of integrating COVID-19 as an SSI into science learning.

### The COVID-19 crisis as an opportunity for SSI exploration

For many countries around the globe, the COVID-19 crisis and governmental emergency response plans disrupted all levels of education during 2020 and 2021 (UNGA, 2021). Widespread emergency school closures meant teachers and pupils were required to rapidly switch from face-to-face (F2F) to emergency remote education (OECD, 2021; UNGA, 2021). This type of education is characterized by online teaching, distance learning, blended learning, and mobile learning during a crisis (Cahapay & Labrador, 2021). Teachers noted adjusting their instructional practices to online and remote education increased workload during emergency school closures (Devitt et al., 2020). They also reported increased technology-supported and student-led learning (Cahapay & Labrador, 2021). Research indicated the overall negative impact of the emergency school closures on various wellbeing and educational aspects of young people’s lives (Bray et al., 2020). When schools were open during the COVID-19 crisis, following emergency school closures, measures were in place to minimize the risk of transmission of the virus within schools and communities. These measures included physical distancing, wearing masks, hand hygiene, respiratory etiquette, frequent cleaning of equipment and teaching aids, and spacing of desks or grouping children (WHO, 2020). However, teachers noted that these measures to limit the spread of COVID-19 created barriers to teaching, learning, and assessment (Chadwick & McLoughlin, 2021). Amidst the COVID-19 crisis, students have the potential to explore this authentic and relevant SSI.

### Addressing the COVID-19 crisis as SSI

The COVID-19 crisis presents an opportunity to explore SSI that are familiar, contemporary, and have clear scientific, societal, moral, and ethical implications (Zeidler & Nichols, 2009). The exploration can be through personal, local, national, and global contexts (OECD, 2019). However, the SSI includes many controversial elements centered on managing a crisis in society (Oulton et al., 2004). The COVID-19 crisis exacerbated students’ exploration of SSI and emergency remote learning posed a heavy workload for the teachers facilitating learning with classroom restrictions (Chadwick & McLoughlin, 2021; Bray et al., 2020; Cahapay & Labrador, 2021). The unprecedented and fast-moving nature of the COVID-19 crisis made worse the lack of resources readily available and designed to address the COVID-19 crisis as SSI (Rosawati & Rahayu, 2021). Addressing the COVID-19 crisis as SSI in science education is timely because of the Irish national educational reform (NCCA 2015; 2018; 2019).

### Science curriculum reform frames SSI exploration

Among other changes in the Irish lower secondary science curriculum, one notable change is the inclusion of an assessment that centers around student inquiry into SSI (NCCA, 2015; 2018). In line with Erduran and Dagher (2014), this research presents an inquiry into teachers embedding SSI exploration within the science curriculum. The research question is framed: *What are secondary science teachers’ perspectives on addressing the COVID-19 crisis as socio-scientific issues?*

## Methodology

### Research context

This study occurred within the Irish education system with secondary science teachers as part of a broader research project exploring their experiences of the COVID-19 crisis in Ireland (Chadwick & McLoughlin, 2021).

### Participants

266 secondary school science teachers participated in this nation-wide study.

### Data collection

The research period was 14 months, between March 2020 and May 2021. The research measures involved online surveys and interviews. The data collected with secondary school science teachers was composed of three phases: Phase I: Online survey (June to July 2020), Phase II: Online survey (December 2020), and Phase III: Online interviews (May 2021). In Phases I and II, the study focused on secondary science teachers’ responses to a single, open-response survey question: *Have you explored coronavirus’s scientific or societal aspects of the COVID-19 pandemic with your students as part of science lessons? For example, students carrying out research/discussion). Please provide details.*

The surveys were sent out to all 5000 secondary school science teachers in Ireland via publicly available school email addresses (Teaching Council, Personal communication regarding the number of registered science teachers in Ireland, 6th July 2021), and promoted through the researchers’ institutional social media channels (Twitter). This represents a response rate of around 5 %.

Phase III consisted of follow-up interviews with five teachers who indicated they had explored COVID-19 as SSI and agreed to contact in relation to the research. Four of the semi-structured interviews, lasting around 30 min, were carried out online, using ‘Zoom’, and audio recorded. One of the five teachers was not available to be interviewed and responded to the interview questions in writing. The interviews asked:

*1. Please describe how you explored coronavirus’s scientific or societal aspects and the pandemic with students in your science classes?*

*2. What were your proposed student learning outcomes/intentions concerning knowledge, skills, attitudes, and values? Did this learning link to the curriculum? Did you gather evidence of student learning relating to these curriculum areas?*

*3. What teaching or pedagogical approaches did you use?*

*4. Did you or your students face any challenges from this approach?*

*5. Did you or your students gain any benefits from this approach?*

*6. Any other comments.*

Qualitative data collection methods were suitable to answer the research question. The typed, open-response survey question quickly and efficiently allowed information gathering from a relatively large number of teachers. We triangulated these findings with findings from semi-structured interviews. These interviews allowed the researchers to probe further on specific issues that may have been missing or limited from the survey (Atkins & Wallace, 2012).

### Data analysis

For developing and representing categories from qualitative data as themes, we used NVIVO software to support the researcher to code, identify, and analyze qualitative information (Braun & Clarke, 2006). According to these authors, this analytical procedure organizes and describes the data in detail and allows for researchers to interpret the data related to the research question. The structural process promotes the validity and reliability of findings that provide an alternative to reliance on the researchers’ direct observations (Creswell & Plano Clark, 2018) during the pandemic.

In this case, inductive thematic analysis provided a detailed account of one specific question relating to an area of interest found in a broader data-set (Braun & Clarke, 2006). We identified themes and sub-themes relating to this area of interest from the survey data and later triangulated against the interview data. We initially explored by reading the data and noting down ideas. We generated initial codes on paper, and where these were related, we collated them into ‘initial’ themes using NVIVO software. We established the final iteration of themes identified from data after the interviews. The themes obtained from the surveys informed the initial coding of interviews. The themes were consistent across the overall data set (surveys and interviews) in terms of the coded references (quotes). Thus, where a relatively large number of coded references (e.g., more than ten) were noted, this defined final themes (Braun & Clarke, 2006). However, it was unnecessary to observe all four final themes in every interview. Table 1 shows the initial themes and the number of references identified from responses from the survey. The final two columns of the table show the renaming and recoding of these initial themes, which progressed to the last themes identified, and the number of references to each final sub-theme. We offer the number of references to each sub-theme to indicate the relative focus on each sub-theme. However, some smaller sub-themes, such as Did explore: Health, wellbeing, and hygiene, were included because they were consistent across the three phases of data collection.

**Table 1:** Progression of initial themes to final themes from Phase I and II surveys

Initial Theme and sub-theme	Number of coded references			Progression of initial to final theme and sub-theme	Number of coded references
	**Phase I**	**Phase II**	**Total**		**Phase I and II**
**Why yes?**				**Did explore**	
The work of scientists	1	4	5	Project work and research	76
Research	25	16	41		
Project work	15	16	31		
Experiment	1	1	2		
Part of the curriculum	42	18	60	As part of the curriculum	60
Discussion	14	27	41	Classroom discussion	41
Wellbeing	2	1	3	Health, wellbeing and hygiene	9
Hygiene	4	1	5		
**Why no?**				**Did not explore**	
Keeping to the curriculum and lack of time	10	9	19	Not part of the curriculum	19
Concerns over student anxiety	9	7	16	Concerns over wellbeing and sensitivity of topic	16
Already too much focus on Coronavirus elsewhere	4	6	10	Already too much focus on the COVID-19 crisis	10
Lack of F2F contact to supervise and support students	4	0	4	Lack of F2F contact (school closures)	7
Lack of confidence	3	0	3		
Plan to/ would like to in the future	10	1	11	*Theme not included (Not informative or descriptive)*	N/A

## Results of survey

Results from surveys (Phase I and II) indicated that 152 out of 266 secondary school teachers (57%) (Phase I and II) stated that they explored the COVID-19 crisis with their students. In comparison, 114 teachers (43%) stated that they did not. The teachers also explained why they did, and did not, explore scientific and societal aspects of the COVID-19 crisis with their students.

**Fig. 1:**
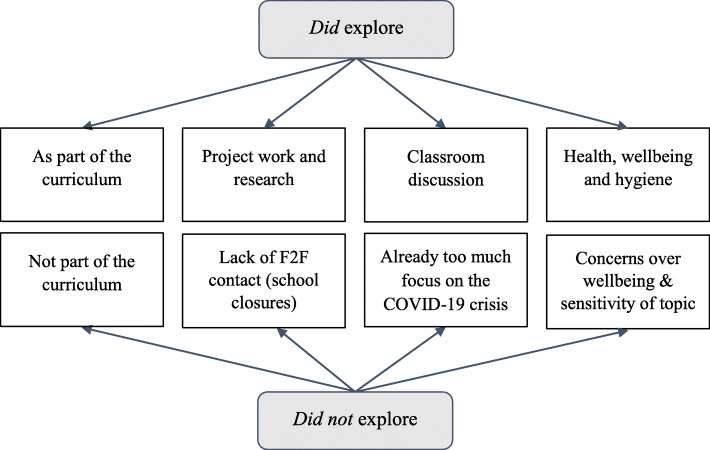
Sub-themes for why teachers *did* and *did not* explore the COVID-19 crisis

Thematic analysis of these survey responses identified four sub-themes for why teachers did explore. Figure 1 shows four sub-themes for why they did not examine the COVID-19 crisis. The reasons why teachers facilitated the exploration of the COVID-19 crisis are as follows: Situated within the curriculum; Explored through project work and research; Expressed through classroom discussion; and benefitted student health, wellbeing, and hygiene (Table 2). Reasons as to why teachers did not explore the scientific and societal aspects of the COVID-19 crisis follow: Not part of the curriculum; the lack of F2F contact for evaluating students’ reactions was challenging; already too much focus on the COVID-19 crisis in everyday life; and concerns over student wellbeing while discussing the sensitive topic of the COVID-19 crisis (Table 2). The teachers discussed how they explored the COVID-19 crisis as part of the science curriculum (Table 2), within particular curricular subjects and topics, e.g., “Microorganisms with first-year” (ST9S1) and “5th Year Biology, in teaching the Immune System” (ST24S1). Some teachers also referred to other curricular subjects such as “English” (ST102S1) and “Maths” (ST135S1 & ST121S2). When talking about project work and research (Table 2), teachers often referred simply to “projects” (ST91S1) and “research projects” (ST51S1), while other teachers described more detail about what they meant by project work and research. For example, a teacher stated, “I produced a report with some false claims that they had to find the false claims and inaccuracies.” (ST106S1). Concerning discussion-based pedagogy, some teachers referred to “discussion” (ST115S1) without contextualizing it (see Table 2). Other teachers reasoned how they facilitated discussion based on a topic (Table 2). Yet, others mentioned discussions centered around students’ “concerns” and “fears” (ST15S2) around the scientific basis of the COVID-19 crisis and societal implications (see Table 2). The teachers described the challenges around facilitating discussion of the COVID-19 crisis. These discussions were mainly saved for F2F teaching, e.g., “[We did not discuss] during our lockdown as I would worry about the students’ fear. We will explore it again in September.” (ST23S1). This sentiment was further confirmed by the higher number of references in the Phase II survey, focusing on F2F teaching, compared to 14 references in the Phase I survey, which focused on the school closures. The teachers mainly focused on discussing how the students could protect themselves from infection when referring to health, wellbeing, and hygiene. For instance, a teacher pointed out she “examined the scientific basis for all the preventative measures such as social distancing and handwashing.” (ST87S1).

**Table 2:** Sub-themes and quotes from Phase I and II surveys

Sub-themes and quotes (Theme: ***did*** explore)	Sub-themes and quotes (Theme: ***did not*** explore)
**As part of the curriculum (60 references):**	**Not part of the curriculum (19 references)**
*With 5th Year Biology, in teaching the Immune System, we discussed Coronavirus in light of how the immune system works as well as how vaccines work. (ST12S1)*	*No time when the curriculum is already difficult to cover under normal circumstances. (ST12S2)*
*Yes, in science and maths we researched and discussed before school closures in March, during school closures and since September. I made sure to cover microbiology and viruses with all students. Covered exponential growth and statistics in maths. We discussed it in SPHE [Social, Personal and Health Education]. (ST121S2)*	*I still stick to the course content - these are not in the learning outcomes. (ST164S1)*
*[I] Supported students to analyze the scientific aspect and concept of how COVID-19 is transmitted and its effect on human body for their English project. (ST102S1)*	*No, not appropriate to physics class. (ST166S1)*
**Project work and research (76 references):**	**Lack of F2F contact (7 references):**
*I have carried out something on immunity and viruses with all of my year groups. It usually involved research on the topic and the creation of PowerPoint slideshows or essays. (ST13S1)*	*We felt as a department it might be better when we are with them just in case they are affected in some way and we didn’t know. This is easier to gauge when you have eye contact/can notice reactions. Parents are also under pressure so we felt it might be seen in a negative light. We will be doing it on return [to F2F teaching]. (ST156S1)*
*Yes, students were asked to do a research piece on any historical pandemic or epidemic of plants or animals - origins, location, symptoms, transmission, treatment* etc. *and staying well. (ST50S1)*	*I didn’t discuss with students as I felt unable to assess how they were coping and if they had been directly affected. (ST95S1)*
*Yes, they have been assigned activities to look in to it more and explain what the virus is, how it spreads and why it affects different groups of people. Some are focusing on it for their SSI [assessment] and the vaccine. I have also got them to explain it as an assignment in TikTok type videos so they have to keep it brief on a time limit but also use diagrams and make it catchy so more people would pay attention to what they say instead of seeing as a long written assignment. (ST99S2)*	
**Classroom discussion (41 references):**	**Too much focus on the COVID-19 crisis (10 references):**
*I discuss breakthroughs as they occur,* e.g. *the vaccine developments. I also pin up newspaper clippings of COVID articles which the students seem to find fascinating by virtue of the fact that they don’t read newspapers or be that familiar with the concept of them. (ST7S2)*	*I have not. I felt it was a topic that was heavily discussed at home, amongst peers and on all media platforms so personally I wanted to take students’ mind off the ongoing crisis during my lessons and give them a mental break by discussing and studying other aspects of science. (ST99S1)*
*We have discussed it in class on numerous occasions. It comes up a lot. As a science teacher, you are on a powerful stage. It is nice to see kids ask questions to get real answers from a science teacher. (ST95S2)*	*Give the students a break from all the talk about it. I was conscious the students were living in the lock down with it going on around them. (ST144S1)*
*In 3rd year science we discussed the impact on the environment of the world-wide shutdown, including the reduction in the levels of air travel, transport in general, production of materials* etc. *We tried t*	
*o think of some positives that may arise due to the circumstances of the measures to try to control the pandemic. (ST155S1)* *Discussion about their concerns (and often, fears) surrounding the pandemic, discussions around viruses and why they aren’t as easily treated as other pathogens, the effect of the pandemic on their social life. (ST15S2)*	
**Health, wellbeing and hygiene (9 references):**	**Concerns over wellbeing and sensitivity of topic (16 references):**
*Yes. Proper handwashing procedures, how germs are spread, germ mutations, the importance of nutrition, exercise, fresh air and sunlight in staying healthy. (ST2S1)*	*I didn’t think it was appropriate. Some students had lost grandparents/family members to COVID-19 and others had parents/family members working in healthcare. I didn’t want to add to their anxiety. (ST135S1)*
*Yes, we looked at viruses and the immune system and examined the scientific basis for all the preventative measures such as social distancing and hand washing. (ST87S1)*	*I don’t want the students to focus on it too much. There is a lot of anxiety amongst students. (ST13S2)*

Teachers explained why they chose not to explore the scientific and societal aspects of the COVID-19 crisis with their students. Some teachers stated that it was not part of the curriculum. An example is, “I followed our scheduled program for the year.”(ST88S1). Others stated that there was no time. For instance, one teacher stated, “No, due to lost class time and trying to finish the syllabus.” (ST86S2). Teachers also described the lack of F2F contact, making it challenging to gauge students’ reactions to challenging topics, e.g., “I felt unable to assess how they were coping.” (ST95S1). The teachers described that there was already too much focus on the COVID-19 crisis in the students’ daily lives and that school should provide a break from these discussions, e.g., “I thought they heard enough and did not need another unnecessary reminder.” (ST68S1). Teachers also voiced their concerns over the impact on students’ wellbeing by discussing this sensitive topic, e.g., “I didn’t think it was appropriate … I didn’t want to add to anxiety.” (ST111S1).

## Results of interview

In Phase III, follow-up interviews were conducted with five teachers who had addressed the COVID-19 crisis as SSI. They discussed why they explored the scientific and societal aspects of the COVID-19 crisis with their students and gave details of how they carried out these lessons. The sub-themes identified in the surveys (see Table 1) were used to support thematic analysis of the interview transcripts to form final themes that were consistent across the entire data set. Four themes were identified from the interviews. They are as follows: As part of the curriculum, Project work and research, Classroom discussion; Health, wellbeing and hygiene.

### Theme 1: as part of the curriculum

The teachers described how they explored the COVID-19 crisis as part of the lower and upper secondary Irish curriculum. The following excerpts reveal how each teacher dealt with the SSI.

*Science, biology, maths, or SPHE (Social, Personal and Health Education). Exponential growth was as a maths concept and the concept in microbiology … they could link what they had learned about exponential growth to the implications for the health system, now for society. The seniors were under pressure, with the Leaving Certificate Biology so even to get a little bit of revision in about DNA and RNA, because these are all the things they were hearing in the media, so I said, kind of a good time to make their biology seem relevant.* (Teacher 1, Interview, 05/29/2021)

Teacher 1 described the subjects where SSI aspects of the COVID-19 crisis could be taught within the prescribed curriculum, including: lower secondary science, upper secondary biology, maths and Social, Personal and Health Education (SPHE). They noted difficulties with the limited time available within the curriculum, with being “under pressure” with the senior biology curriculum. They resolved this by identifying the aspects of the course that were “relevant” to learning about the COVID-19 crisis.

*We did it with the seniors on their [biology] course. They actually have to study the virus, and how viruses work. Not that it's on the curriculum for the first years but I did do it with first years because it's society, and how science works.* (Teacher 2, Interview, 05/28/2021*)*

Teacher 2 identified that SSI aspects of the COVID-19 crisis could be taught within upper secondary biology curriculum, namely learning about “the virus, and how viruses work”. Furthermore, they described their choice to address the COVID-19 crisis with first year students in lower secondary science, despite it not being “on the curriculum”. Teacher 2 took the opportunity to explore SSI with these students because it was related to the relevant issue of societal implications of science.

*I let them explore what a virus was earlier than we normally would have, whereas normally you would do microbiology later on in second year or third year. The challenge was that we were getting very little of anything else covered … but I think it was worth it. I have an Agricultural science class, fifth and sixth year. They were very interested in the vaccine and interested in what's going to happen next., interested in the pandemic globally … Linking it to zoonotic and notifiable diseases.* (Teacher 3, Interview, 05/24/2021)

Teacher 3 described changing the order in which topics were taught to allow for exploration of the COVID-19 crisis. They did this by encouraging students to “explore what a virus was earlier than we normally would have” in lower-secondary science classes. They incorporated the COVID-19 crisis into upper secondary Agricultural Science classes by “linking it to zoonotic and notifiable diseases”. They noted difficulties with addressing the COVID-19 crisis within the time available within the science curriculum, stating that they were “getting very little of anything else covered”. However, Teacher 3 noted that the benefits outweighed the drawback, stating that “it was worth it”.

*I didn’t have a biology group, I had chemistry … give them the background science in a way that's more chemistry focused just to bring in the usefulness of chemistry. Sometimes when you're teaching a senior group you're just trying to hammer through the course.* (Teacher 4, Interview, 05/27/2021)

Teacher 4, a chemistry teacher, stated that they incorporated exploration of SSI into upper secondary chemistry classes by giving them the background science of the COVID-19 crisis in a “chemistry focused” way. They noted time pressures within the senior curriculum, therefore “trying to hammer through the course”.

*5th year Biology: Applied the Scientific Method to COVID-19. 6th year Biology: Discussed COVID-19 when studying the virus chapter. Assessing student learning as per the learning outcomes of the syllabus.* (Teacher 5, Interview (written response via email), 05/24/2021)

Teacher 5’s stated that they explored the SSI aspects of the COVID-19 crisis in upper secondary biology while applying the scientific method and learning about viruses. They were able to assess student learning as part of this by following the learning outcomes of the curriculum.

#### Summary

The five teachers stated that they explored the COVID-19 crisis as part of the lower and upper secondary Irish curriculum. The teachers described how they identified where SSI aspects of the COVID-19 crisis could be taught within the prescribed curriculum in a range of subjects including: lower secondary science; upper secondary biology, chemistry and agricultural science; mathematics; and SPHE. They described changing the way or the order in which topics were taught to allow for timely exploration of the COVID-19 crisis. Teachers noted difficulties with addressing the COVID-19 crisis within the limited time available for covering the curriculum and this was particularly apparent in upper secondary level science. For example, “under pressure” (Teacher 1), “trying to hammer through the course” (Teacher 4). Teacher 2 described their choice to address the COVID-19 crisis despite it not being on the curriculum. However, Teacher 3 indicated that the benefits of exploring the COVID-19 crisis with students outweighed the negatives.

### Theme 2: project work and research

Four out of the five teachers interviewed described how they facilitated students to explore the COVID-19 crisis through project work and research, in a variety of ways. Teacher 2 expressed that they did not facilitate project work or research. The following excerpts reveal how each teacher dealt with the COVID-19 crisis as SSI.

*There was a lot of fake news … so really we need to get the science of this and give them the tools so that they could understand some of what was happening … sending them off to do a little bit of research and write a summary about what you found, to get that kind of digital literacy and where to go for good information.* (Teacher 1, Interview, 05/29/2021)

Teacher 1 described facilitating students to “research” the scientific and societal aspects of the COVID-19 crisis. Teacher 1 described this as giving the students the “tools” in “digital literacy” to empower them to “understand some of what was happening”, and critical skills to be able to identify “fake news”.

*No, I didn't go down the project route. Some teachers will have students making posters and projects about COVID-19. For me, the thought of sitting down and doing a project on it, I just didn't see that they’d have any interest. No, just the classroom discussion.* (Teacher 2, Interview, 05/28/2021)

Teacher 2 stated that they did not facilitate project work or research on the SSI aspects of the COVID-19 crisis. They noted that they “didn’t go down the project route” and justified this by noting that they didn’t think the students would “have any interest”. Instead, teacher 2 facilitated “classroom discussion”.

We were looking up media reports to see where to get your information. I gave them the websites. I said this is where you get your information from, don't be looking just anywhere. So they took it all done in their journals. I was saying to them was that helpful? Did you look up anything? Did you find out anything?

We did an experiment. They put oil on their hands and they try to wash it off with cold or warm or soapy water or hand sanitizer. We did a little bit 1of work around that and figuring out why it works*.*

*They learned the scientific method. They learned about society. They learned how science and society works for their benefit. They'll be able to apply that knowledge to anything going forward.* (Teacher 3, Interview, 05/24/2021)

Teacher 3 gave details about how they facilitated project work and research in a variety of ways. Teacher 3 described facilitating students to research by “looking up media reports” and “websites” that were reliable rather than “looking just anywhere”. As part of project work, Teacher 3 described facilitating an “experiment” comparing how effectively oil can be removed from hands using “cold or warm or soapy water or hand sanitiser”, “figuring out why it works”, and comparing this to methods of reducing transmission of the COVID-19 virus. Teacher 3 noted that they focused on “the scientific method” and how students would be able to apply the scientific method to “anything” relating to the COVID-19 crisis in society going forward.

*At points where there would have been a breakthrough, occasionally, I might have thrown up the PowerPoint with a little bit of information or reference to one or two good articles, and I would have referenced quite a bit … or I might send them a link to an article through Google classroom.* (Teacher 4, Interview, 05/27/2021).

Teacher 4 briefly described facilitating students to engage with research on the COVID-19 crisis by providing examples of “good articles” for students to engage with.

*Applied the Scientific Method to COVID-19 / Understand the steps of the Scientific Method with a real life current example; [Exploring] studies from the Internet; Information about COVID-19 was subject to change and students needed an awareness of this.* (Teacher 5, Interview (written response via email), 05/24/2021)

Teacher 5 described encouraging students to engage with research on the COVID-19 crisis, e.g. “studies from the internet”. They also described how they encouraged students to apply the scientific method to the COVID-19 crisis by understanding the “scientific method with a real life current example”. They noted that they promoted student awareness that due to the fast paced nature of this real life example, information about the COVID-19 crisis “was subject to change”.

#### Summary

Four of the five teachers described how they facilitated students to explore the COVID-19 crisis through project work and research, in a variety of ways. Teachers 1, 3, 4 and 5 described facilitating students to conduct “research” (Teacher 1), e.g. “looking up media reports” (Teacher 3) or engage with research on the COVID-19 crisis, e.g. “studies from the internet” (Teacher 5). However, Teacher 2 stated that they did not “go down the project route” (Teacher 2). Teacher 3 described facilitating an “experiment” comparing how effectively oil can be removed from hands using “cold or warm or soapy water or hand sanitiser” and “figuring out why it works” (Teacher 3).

### Theme 3: classroom discussion

All five teachers described how they used classroom discussion as a way to explore the scientific and societal aspects of the COVID-19 crisis. The following excerpts illustrate how the teachers explored the SSI.

*We had loads of informal discussion about what was happening. There was a lot of spontaneous, directed class discussion … The biggest challenge I found as a teacher was managing discussion. So, when it would start to kind of go into fake news, and even racism, and stuff like that. So just trying to keep a lid on, anything that was offensive.*

*I mainly saved the class discussion for when we returned face to face. And it was easier. We had some discussions online but it was easier to have the challenging discussions when we were in school.* (Teacher 1, Interview, 05/29/2021)

Teacher 1 described including “informal” and “spontaneous” discussion about the COVID-19 crisis. They described their main challenge as “managing discussion”, balancing the need for open discussion with limiting “offensive” comments, giving examples such as “fake news” and “racism”. For these reasons, Teacher 1 indicated that while they did have some discussions online during emergency school closures, they mainly conducted these discussions during F2F teaching.

*They were obviously very interested, and it came across they actually wanted to know more … then I genuinely stopped doing any more teaching and asked them for their questions, because they had absolutely loads … I went along the approach to drive their conversations as part of the lesson that I would teach … I let them lead us, which is probably why they were so interested in it, which would be more different than I would normally do … They were quite open with their conversation … The benefit I can see was that they understood more of what was actually happening in the world.* (Teacher 2, Interview, 05/28/2021)

Teacher 2 described making time for class discussion, based around students leading the direction of conversation, by allowing students to voice their questions rather than direct teaching. Teacher 2 noted that this benefited the students by increasing their understanding of the COVID-19 crisis globally.

*Trying to get that information out there to them, to show them that they can have some control and some impact. I would discuss the numbers with them, nearly daily, when the numbers were coming out, daily, of infections.* (Teacher 3, Interview, 05/24/2021)

Teacher 3 included daily discussion of the COVID-19 crisis in their lessons. Their aim was to provide information and increase students’ understanding of the numbers of infections. This worked to empower students to feel more in control of the situation and value their personal ‘impact’.

I approached it a little bit more informally. So anytime there would be a major event related to the science, vaccine or something from government, I would give a few minutes in class to discuss. They wanted to discuss the issues and some of them were actually quite blunt about how the government was managing things and how they felt society was or wasn't doing things correctly … They were unsure of the science and were looking for clarification from a trusted source … Having a more rational scientific discussion about what's factual, what’s scientific and just getting used to the idea of scientifically judging information as it comes across and we would have a little bit of discussion*.*

*I didn't want the students to feel this was something that had to be correct or incorrect. I wanted the discussion to be a bit more free-flowing. In the discussion I may pick up, in a pastoral sense, what was going on. They're not getting the opportunity at home, because maybe their parents aren’t scientifically inclined and maybe they're hearing so many different things from different sources.* (Teacher 4, Interview, 05/27/2021).

Teacher 4 described in detail how they facilitated informal discussion relating to the COVID-19 crisis. They noted that each time new developments arose in the fast-paced and contemporary SSI context they would facilitate a discussion, i.e., “Anytime there would be a major event”. Teacher 4 discussed aspects of “the science”, government responses, development of the vaccine, how society in general was responding to the crisis. They aimed to focus on “free-flowing”, “rational, scientific discussion” while also encouraging students to appreciate the nuance of the situation, i.e. not “correct or incorrect”. Teacher 4 noted that one benefit for students was to get their information from a “trusted source” and “clarification” of the science. This worked to improve students’ capacity to judge the information while they were exposed to “so many different things from different sources”. For Teacher 4 noted that they were able to “pick up, in a pastoral sense, what was going on” with the students and monitor students’ wellbeing through these discussions.

*Discussed COVID-19 when studying the virus chapter. Scientific theory explained through a real life current example.* (Teacher 5, Interview (written response via email), 05/24/2021)

Teacher 5 briefly discussed classroom discussion of the COVID-19 crisis. They noted that the discussions were linked to the biology curriculum area of viruses and they used the real-life example of the COVID-19 virus.

#### Summary

All five teachers described how they used classroom discussion as a way to explore the scientific and societal aspects of the COVID-19 crisis. This included “informal”, “spontaneous” (Teacher 1) discussion; answering students’ questions; and discussions of related curricular content, e.g. “when studying the virus chapter” (Teacher 5). Teacher 2 described challenges with facilitating these discussions, e.g. “The biggest challenge I found as a teacher was managing discussion … just trying to keep a lid on, anything that was offensive” (Teacher 1), “Students … relatives that would have had the virus … parents that are extremely ill. I suppose you had to bear that in mind and not go overboard.” (Teacher 2). Teacher 1 indicated that they mainly conducted these discussions during F2F teaching, rather than online due to the challenging nature of these discussions.

### Theme 4: health, wellbeing and hygiene

The teachers who were interviewed discussed how exploration of the COVID-19 crisis as SSI in their science classes contributed positively to students’ health, wellbeing and hygiene. The following excerpts reveal how these teachers dealt with the SSI.

*They were very stressed about the big unknown and [we were] just trying to have a discussion … well this is what the science knows.* (Teacher 1, Interview, 05/29/2021)

Teacher 1 briefly discussed how the COVID-19 crisis had impacted students’ wellbeing causing them to be “very stressed”. They attempted to reassure students by “trying to have a discussion” about “what the science knows”.

*Deeper understanding of the reasons why we want them to wash their hands and not party and gather in groups.*

*Students in the room had relatives that had the virus and parents that are extremely ill. I suppose you had to bear that in mind and not go overboard. I think the biggest thing for the students was, they just felt so alone. You can actually see it on them, just the despair, when is this going to end and is there an ending? I was trying to give them the positivity that one day we will get past this and get through this.* (Teacher 2, Interview, 05/28/2021)

Teacher 2 discussed the negative impact on the students’ wellbeing, referring to students feeling isolated (“alone”) and in “despair”. Teacher 2 aimed to give students a more positive outlook and provide hope that “that one day we will get past this and get through this”. Teacher 2 also noted benefits to students’ hygiene by developing their understanding of the “the reasons why we want them to wash their hands” in terms of transmission of the COVID-19 virus. Teacher 2 also aimed to increase students’ understanding of the rationale behind physical distancing in schools and society (“not party and gather in groups”).

*I think they understood it more and it took the fear out of it. We were saying, what can you control? It feels like it's all out of our control, but which part of this is in our control. So, washing our hands, cleaning our desk.* (Teacher 3, Interview, 05/24/2021)

Teacher 3 discussed how they empowered students to feel more in “control” and take some of the “fear” away by encouraging them to see what steps they could take to protect themselves against COVID-19 infection (“washing our hands, cleaning our desk”).

I was cautious about overdoing anything on COVID because the students are getting saturated with it … I didn't want to create an echo chamber in the classroom where their worries amplified … I think the students felt a little bit more relaxed about discussing it*.*

*Fully explaining why hand washing and sanitizing were really, really important … We had quite a bit of info about risk and assessing risk.* (Teacher 4, Interview, 05/27/2021).

Teacher 4 aimed to take a “cautious” approach to exploring the COVID-19 crisis. They felt that students were already overly exposed to talk about COVID-19 and that there was a risk of creating more worry amongst the students. However, teacher 4 noted that the students benefited from discussion of the COVID-19 crisis by feeling “a little bit more relaxed”. They also felt that students’ understanding of the importance of hygiene in relation to virus transmission was improved, referring to “hand washing and sanitizing were really, really important”.

Teacher 5 did not discuss the theme Health, wellbeing and hygiene.

#### Summary

The teachers who were interviewed discussed the negative impacts of the COVID-19 crisis on students’ wellbeing. They noted how exploration of the COVID-19 crisis as SSI in their science classes contributed positively to students’ health, wellbeing and hygiene. They discussed benefits to students’ wellbeing e.g., “Students felt a little bit more relaxed about discussing it” (Teacher 4) and removed some of the “fear” (Teacher 3). They noted that by exploring the COVID-19 crisis students had improved their understanding of the importance of hygiene and sanitization, empowering students to better protect themselves against virus transmission (WHO, 2020).

## Discussion

Student exploration of SSI is a prime method of developing skills, knowledge, and attitudes relating to scientific literacy (Zeidler et al., 2019). The COVID-19 crisis presents an opportunity for students to explore an authentic, controversial SSI with clear scientific and societal implications within personal, local, national, and global contexts (OECD, 2019; Zeidler & Nichols, 2009). For example, Teacher 1 observes, “It was a great teaching opportunity if you ignore all the sickness and death.” However, during the COVID-19 crisis, emergency school closures and restrictions to in-classroom teaching negatively impacted teachers’ capacity to facilitate learning, increased teacher workload, and adversely affected student wellbeing and learning (Bray et al., 2020; OECD, 2021; UNGA, 2021).

During the COVID-19 crisis, this study aimed to explore secondary level science teachers’ perspectives on addressing the COVID-19 crisis as SSI. In this study, 57% of secondary-level science teachers surveyed indicated that they explored the COVID-19 crisis with their students. Teachers outlined four main barriers to exploring the COVID-19 crisis (Fig. 1): 1. The COVID-19 crisis was not part of the curriculum; 2. The lack of F2F contact made judging students’ reactions challenging; 3. There was already too much focus on the COVID-19 crisis in everyday life; 4. Concerns over student wellbeing while discussing the sensitive topic of the COVID-19 crisis. However, teachers who explored the COVID-19 crisis with their students showed how they overcame these barriers by exploring it: 1. within the curriculum by identifying where it could be taught and changing plans where necessary; 2. through project work and research, F2F, and during emergency school closures; 3. through F2F classroom discussion; 4. This led to benefits to student health, wellbeing, and hygiene. In keeping with the findings from this study, wider literature describes a range of challenges for teachers who choose to explore SSI with their students (Bayram-Jacobs et al., 2019; Chen & Xiao, 2020). These include lack of time within the curriculum and higher prioritization of content knowledge acquisition, e.g. “No time as I had exam classes” (ST44S1), “I still stick to the course content - these are not in the learning outcomes.” (ST164S1). A lack of readily available materials for teaching SSI often means heavy workload due to planning, organization and classroom management considerations. These challenges are exacerbated due to the need for specific pedagogical skills and knowledge for managing exploration of potentially upsetting SSI with their students (Chen & Xiao, 2020; McCully et al., 1999), e.g. “Students are deeply affected by the current situation … I don’t like to make them dwell on it or feel upset about it more than they already are.” (ST128S1). The COVID-19 crisis increased these time and workload pressures on teachers (Chadwick & McLoughlin, 2021; Devitt et al., 2020). In addition, at times during the COVID-19 crisis teachers were facilitating emergency remote education with their students, making the instructional management of discussion, research and project work, based on the COVID-19 crisis, more challenging due to the lack of F2F contact with students (Cahapay & Labrador, 2021). “It might be better when we are with them, just in case they are affected in some way and we did not know. This is easier to gauge when you have eye contact/can notice reactions.” (ST145S1). Despite these challenges, the majority of teachers in this study addressed the COVID-19 crisis as SSI. This supports and extends recent research, which indicated teachers’ positive intentions and confidence towards exploring the COVID-19 crisis with their students (Rosawati & Rahayu, 2021).

Teachers in this study who did not explore the COVID-19 crisis stated that it did not fit in with the prescribed curriculum and there was no time available to do so (Chen & Xiao, 2020). Teachers noted that time limitations were particularly apparent in the senior secondary curriculum. For example, Teacher 4 stated “When you’re teaching a senior group you’re just trying to hammer through the course”. However, teachers who did explore the COVID-19 crisis circumvented the perceived limitations of the curriculum and overcame this barrier by identifying where aspects of the COVID-19 crisis could be taught within the existing curriculum in a range of subjects including science, biology, chemistry, maths, English and SPHE curricula (Table 2). Teachers also changed their plans to teach topics that were more relevant to the COVID-19 crisis while still following the prescribed curriculum, “In any aspect of the curriculum where the physical/ chemical/ biological/ mathematical nature of COVID-19 may be applied to the lesson.” (ST84S2). Notably, some teachers who did address the COVID-19 crisis as SSI acknowledged that it did not fit within the curriculum but did so regardless, due to perceived benefits to students, e.g., “Not that it’s on the curriculum for the first years but I did do it.” (Teacher 2 interview). Teacher 3 in their interview stated, “we were getting very little of anything else covered … but I think it was worth it.”.

Teachers who facilitated student exploration of the societal and scientific aspects of the COVID-19 crisis mainly used two teaching approaches: Project work and research, and classroom discussion. Recent research suggests that carefully planned learning experiences addressing SSI can develop both curricular content knowledge and a range of skills and attitudes contributing to scientific literacy (Zeidler et al., 2019). Teachers in this study described using “projects” (ST90S2) and “research” (ST85S2) to develop skills typically associated with exploration of SSI, including critical evaluation, e.g., “Scientifically judging information” (Teacher 4); and evaluating the value and limitations of science and technology, e.g., “the development of new technologies can actually help science. The research behind it and how much research that would have had to be done.” (Teacher 2). Development of these skills requires sustained engagement with SSI learning experiences, rather than one-off activities (Zeidler et al., 2019). In this study, teachers indicated sustained engagement with the COVID-19 crisis as SSI, through regular discussions with students. Teacher 3 in the interviews noted, “I would discuss the numbers with them, nearly daily.” Another teacher provided opportunities for students to ask questions: “questions are encouraged every day as the COVID-19 crisis progressed.” (ST114S2). Exploration of controversial SSI has the potential to cause conflict and upset within the classroom. The onus is on the teacher to carefully manage such situations so teachers who do explore controversial SSI must show strong commitment, enthusiasm and willingness to take risks (McCully et al., 1999).

Findings from this study described teachers’ concerns around student wellbeing, stress and anxiety. These were barriers to addressing the COVID-19 crisis as SSI and how the lack of F2F contact during emergency school closures meant that it was difficult to judge in-person how students were responding to activities and discussions (Cahapay & Labrador, 2021). In overcoming these barriers, teachers mainly conducted COVID-19 crisis discussion in person (F2F), whilst schools were open. Teachers noted that by exploring the COVID-19 crisis, they contributed positively to student health, wellbeing, and hygiene, countering some of the crisis’ negative impacts on students (Bray et al., 2020; UNGA, 2021). Teachers stated that students were more relaxed about discussing the COVID-19 crisis and had less fear. Teachers also noted benefits relating to educating students about staying healthy during the COVID-19 crisis and minimizing their risk of infection.

## Conclusion

The findings of this study support and extend recent research (Bayram-Jacobs et al., 2019; Zeidler et al., 2019) around the challenges of addressing SSI in a sustained and long-term way within secondary level curricula. Science teachers in this study described barriers to addressing SSI within the secondary curriculum. These findings align with existing research (Bayram-Jacobs et al., 2019), noting challenges around time and resources available for SSI-based instruction within existing curricular frameworks. In Ireland, changes to the lower secondary science curriculum emphasize the exploration of SSI, including the addition of an SSI-based assessment (NCCA, 2015; 2018). Within this context, most Irish secondary science teachers surveyed indicated that they had addressed the COVID-19 crisis as SSI. This positive response highlights the need to embed and emphasize SSI exploration within secondary-level science curricula, creating the time and flexibility needed for SSI exploration. The COVID-19 crisis presents the ultimate, authentic SSI due to its personal, local, national, and global implications (OECD, 2019). While recent research noted that teachers planned to explore the COVID-19 crisis as SSI and felt confident doing so (Rosawati & Rahayu, 2021), this study went a step further by asking teachers for details of *how* they had addressed the COVID-19 crisis in practice. The majority of teachers in this study facilitated SSI exploration, despite additional challenges stemming from the COVID-19 crisis. This claim concurs with the findings of Cahapay and Labrador (2021), Chadwick and McLoughlin (2021), Devitt et al. (2020). The result concerning the general barriers to exploring SSI supports Bayram-Jacobs et al. (2019) and McCully et al. (1999). Importantly, teachers in this study noted that addressing the COVID-19 crisis as SSI led to benefits to student well-being, going some way to counteract the negative impacts of the COVID-19 crisis on student learning and wellbeing (Bray et al., 2020).

### Implications

While some science teachers in this study described barriers to addressing SSI within the Irish secondary science curriculum, the majority of participating teachers did explore the COVID-19 crisis as SSI within the curricular frameworks. This is likely in part due to the recent inclusion of SSI-based assessment within the lower-secondary science curriculum, affording the level of curricular flexibility needed for authentic SSI exploration (NCCA, 2015; 2018). This highlights the need to continue to emphasize the exploration of SSI within the secondary science curriculum in Ireland. Specifically, this progress may be continued with the integration of SSI based teaching, learning and assessment in the upper-secondary science (biology, chemistry, physics and Agricultural science) curriculum in Ireland, which is currently under review (NCCA, 2019). This may go some way to address teachers’ concerns around time limitations, by stipulating time within the curriculum for exploration of relevant and timely SSI.

This study highlights teachers’ willingness to engage with SSI based teaching and learning within the secondary school science curriculum. Furthermore, teachers saw the benefit of SSI based instruction to their students in counteracting some of the negative impacts of the COVID-19 crisis. This enthusiasm can be harnessed and built upon through targeted continuous professional development activities for teachers that continue and extend the good practice identified in this study. This professional development can be used to widen the practice identified by highlighting the relevance and flexibility of SSI based learning in a variety of arising contexts in our contemporary world (Zeidler et al., 2019).

### Limitations of the study

The study is qualitative with a limited number of participants. It does not aim to provide a representative sample of the approximately 5000 secondary level science teachers in Ireland (Teaching Council, Personal communication regarding the number of registered science teachers in Ireland, 6th July 2021). Teachers voluntarily responded to online surveys and interviews. Responding online was not a problem because all secondary teachers have access to the internet while in school (DES, 2015). However, the teachers who completed the surveys are likely interested in the topic explored. We carried out a small number of interviews with those teachers who firstly indicated that they had studied the scientific and societal aspects of the COVID-19 crisis and were sufficiently motivated about the subject matter to take the time to participate in an interview (Atkins & Wallace, 2012). Therefore, the authors acknowledge the limitations of this research about the scope and transferability (Atkins & Wallace, 2012). This study aims to provide critical insights into the research question. Recommendations originate from the teachers’ perspectives presented in this study and are rooted in the broader literature. This low-risk study received ethical approval from the Research Ethics Committee at the authors’ institution, and all teachers gave written consent to participate.

## Data Availability

The datasets used and/or analysed during the current study are available from the corresponding author on reasonable request.

## Abbreviations

COVID-19: Coronavirus disease 2019
F2F: Face-to-face
OECD: Organisation for Economic Cooperation and Development
SPHE: Social, Personal and Health Education
SSI: Socioscientific Issues
WHO: World Health Organisation
